# The impact of chronic stress on intracellular redox balance: A systems level analysis

**DOI:** 10.14814/phy2.15640

**Published:** 2023-04-05

**Authors:** Hannah Geddie, Megan Cairns, Logan Smith, Minette van Wyk, Leandrie Beselaar, Nina Truter, Fanie Rautenbach, Jeanine L. Marnewick, Danzil E. Joseph, M. Faadiel Essop

**Affiliations:** ^1^ Center for Cardio‐metabolic Research in Africa (CARMA), Department of Physiological Sciences Stellenbosch University Stellenbosch South Africa; ^2^ Applied Microbial and Health Biotechnology Institute (AMHBI) Cape Peninsula University of Technology Cape Town South Africa; ^3^ Centre for Cardio‐metabolic Research in Africa (CARMA), Division of Medical Physiology, BMRI, Faculty of Medicine and Health Sciences Stellenbosch University Cape Town South Africa

**Keywords:** brain, chronic stress, heart, liver, oxidative stress, UCMS model

## Abstract

Chronic psychosocial stress is implicated in the onset and progression of noncommunicable diseases, and mechanisms underlying this relationship include alterations to the intracellular redox state. However, such changes are often investigated in isolation, with few studies adopting a system level approach. Here, male Wistar rats were exposed to 9.5 weeks of chronic unpredictable mild stress and redox status assays were subsequently performed on cardiac, hepatic, and brain tissues versus matched controls. The stressed rats displayed an anxious phenotype, with lowered plasma corticosterone levels (*p* = 0.04 vs. Controls) and higher plasma epinephrine concentrations (*p* = 0.03 vs. Controls). Our findings showed organ‐specific redox profiles, with stressed rats displaying increased myocardial lipid peroxidation (*p* = 0.04 vs. Controls) in the presence of elevated nonenzymatic antioxidant capacity (*p* = 0.04 vs. Controls). Conversely, hepatic tissues of stressed rats exhibited lowered nonenzymatic antioxidant capacity (*p* < 0.001 vs. Controls) together with increased superoxide dismutase (SOD) activity (*p* = 0.05 vs. Controls). The brain displayed region‐specific antioxidant perturbations, with increased SOD activity (*p* = 0.01 vs. Controls) in the prefrontal cortex of the stressed rats. These findings reveal distinct stress‐related organ‐specific vulnerability to redox perturbations and may provide novel insights into putative therapeutic targets.

## Graphical abstract

Chronic psychosocial stress is implicated in the onset and progression of non‐communicable diseases, and mechanisms underlying this relationship include alterations to the intracellular redox state. Here, male Wistar rats were exposed to 9.5 weeks of chronic unpredictable mild stress and redox status assays were subsequently performed on cardiac, hepatic and brain tissues versus matched controls. Our findings reveal distinct stress‐related organ‐specific vulnerability to redox perturbations and may provide novel insights into putative therapeutic targets.

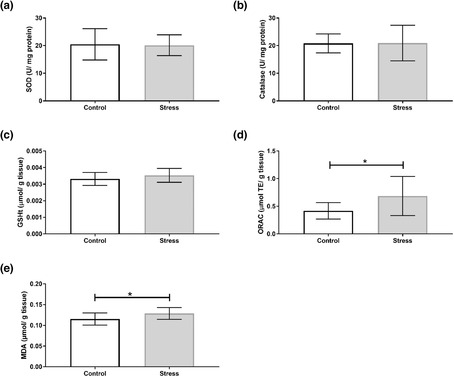

## 1 INTRODUCTION

Chronic psychological stress is a major risk factor for several noncommunicable diseases ranging from neuropsychiatric to cardiometabolic complications (Ghosh & Verma, 2018). This relationship is underpinned by robust evidence and points toward a dysfunctional stress response as the primary causative factor (McEwen, 2008). The physiological stress response comprises the hypothalamic–pituitary–adrenal axis (HPA) and the sympathoadrenal‐medullary pathway (SAM) (Sher et al., 2020). These systems work in concert to release hormones and neurotransmitters responsible for both the rapid fight‐or‐flight response and the longer‐acting effects of cortisol. While such pathways are integral to human survival within the acute setting, excessive or prolonged mediator release can disturb homeostasis and trigger pathophysiologic outcomes (McEwen, 2017).

Although the precise mechanisms underlying the shift from beneficial to pathological stress remain unclear, the evidence suggests that prolonged exposure to stress mediators can negatively impact the intracellular redox balance (Aschbacher et al., 2013). In support, several studies implicate oxidative stress as a key driver of chronic stress pathology in a variety of organs (Ishtiaq et al., 2018; Li & Xia, 2020; Rabasa & Dickson, 2016; Schiavone et al., 2013). Of note, most oxidant production is not inherently detrimental, as such molecules play a key role in physiologic intracellular signaling circuits (Finkel, 2012). In contrast, oxidative stress refers to the disequilibrium between oxidant production and antioxidant capacity and is characterized by irreversible changes to the structure and function of numerous important intracellular components (Sharifi‐Rad et al., 2020). Such changes can lead to harmful alterations/ modifications in intracellular signaling cascades, impacting overall cell function to eventually lead to the activation of cell death pathways (Battistelli et al., 2016; Xu et al., 2019a).

Maintenance of intracellular redox balance is therefore a tightly regulated and dynamic process. The cell continuously adjusts antioxidant transcription to balance its energy and signaling demands against the need to prevent oxidative damage (Patel et al., 2014; Raghunath et al., 2018). Oxidative stress can therefore result from an increase in oxidant production, a decrease in antioxidant capacity, or a combination of both (Pizzino et al., 2017). Interestingly, antioxidant profiles and susceptibility to oxidative damage can differ between cell types, and hence mechanisms linking psychological stimuli to systemic oxidative stress are likely of a complex nature and manifest in an organ‐specific manner (Kim et al., 2018).

Despite the clear associations between psychological stress and oxidative stress, there are limited studies that investigate antioxidant and prooxidant parameters in this instance. In light of this, the current study investigated an array of redox parameters in the heart, brain, and liver in a rat model of unpredictable chronic mild stress (UCMS).

## 2 METHODS

### 2.1 Animals

Ten‐week‐old male Wistar rats (~290 g) (Stellenbosch University's Faculty of Medicine and Health Sciences, Tygerberg, South Africa) were housed individually in the Animal Research Facility, located at the university's main campus (Stellenbosch, South Africa). Animals were provided with ad libitum access to standard rodent chow and water while maintaining a usual 12‐h dark/light cycle. This study was conducted with the approval of the Animal Ethics Committee of Stellenbosch University (South Africa) (Ethics #6311), and all handling and procedures were performed in accordance with the Guidelines for the Care and Use of Laboratory Animals of the National Academy of Science (NIH publication no. 85‐23, revised 1996).

### 2.2 Unpredictable chronic mild stress

The rodent model used in this study was adapted from protocols for the UCMS as described previously (Isingrini et al., 2011). Following a 4‐week habituation period, rats were matched according to body weight and sucrose preference and randomly divided into Control (*n* = 14) and Stress (*n* = 14) groups. Of note, due to limited sample availability we could not always employ all the samples for all the different tests/analyses here completed. Thus, our sample values will vary in the data presented. The Stress group was subjected to 9.5 weeks of unpredictable mild stressors (Table 1), which were applied singularly or in tandem, 6 days per week, during the light phase to enhance intensity and unpredictability. Each individual stressor was only applied once per week, and the order of exposure altered with each week of the protocol. Stress and Control animals were separated during stressor application as well as for an additional 4 h (post stressor[s]) to prevent stress pheromones from influencing the Control group.

**TABLE 1:** Summary of stressors applied during the experimental protocol.

Stressor	Description	Exposure length
Damp bedding	Approximately 200 mL water was added to the bedding of each cage.	4 h
Cage tilt	Cages were tilted at a 45° angle.	4 h
Light/dark reversal	Lights in the stress procedure room were intermittently turned on and off every 30 min.	8 h
Social stress	Rats were removed from their cage and made to spend the duration of this stressor in a neighboring rat's cage.	4 h
Predator scent and sound	Concentrated bobcat urine (The Pee Mart, Vassalboro, ME, USA) was added to each cage and a recording of cat sounds was projected throughout the room.	4 h
No bedding	All bedding was removed from the cages.	4 h
White noise	A recording of white noise set to 80 dB was projected throughout the room.	4 h
Strobe light	A strobe light was placed in the stress procedure room.	4 h
Restraint	Rats were placed into Perspex restraint cages (6 cm × 7 cm × 18 cm), specifically designed to limit movement while still allowing them to feel the body heat of their neighbors.	30 min

### 2.3 Euthanasia, blood, and tissue collection

Rats were euthanized via rapid decapitation and heart, liver, and brain tissues were dissected out, washed in ice‐cold 0.1 M phosphate‐buffered saline (pH 7.4, Sigma‐Aldrich, St. Louis, MO, USA), cut into smaller pieces, snap‐frozen in liquid nitrogen, and subsequently stored at −80°C for future analyses. The brain was further dissected to isolate the hippocampus, cerebellum, and prefrontal cortex and thereafter stored in the same manner as before. Trunk blood was collected via a 0.17 M ethylenediaminetetraacetic acid (EDS985, Sigma‐Aldrich) coated glass funnel, directly into ethylenediaminetetraacetic acid vacutainers. Blood was centrifuged at 1, 400 × g for 30 min, after which the plasma was divided into 100 μL aliquots, snap‐frozen in liquid nitrogen, and stored at −80°C.

### 2.4 Enzyme‐linked immunosorbent assays

Plasma corticosterone (E‐EL‐0160; Elabscience®), adrenocorticotropic hormone (ACTH) (#ab263880; Abcam), and epinephrine (E‐EL‐0045; Elabscience®) were assessed via various enzyme‐linked immunosorbent assay (ELISA) kits. All ELISA kits were prepared according to kit instructions with sample dilutions made where applicable. All samples and standards were loaded in duplicate.

### 2.5 Tissue sample preparation

All collected tissue samples were homogenized in 30‐s intervals and sonicated for 10 s at 10 A. Samples were subsequently centrifuged at 15,000 × g for 10 min at 4°C, and supernatants were extracted.

### 2.6 Oxidative stress

Oxidative stress assay samples were prepared in either 1:9 iced 0.1 M phosphate‐buffered saline, pH 7.4 or assay‐specific buffers. Protein content was determined via a Direct‐Detect® infrared spectrometer or a Bradford assay before storage at −80°C. Multiple oxidative stress assays were performed to assess the redox status of the collected cardiac, hepatic, and brain tissues. Catalase, superoxide dismutase (SOD), oxygen radical absorbance capacity (ORAC), as well as reduced and oxidized glutathione assays provided insight into the nature of antioxidant defenses, whereas the thiobarbituric acid reactive substances (TBARS) assay highlighted the presence of oxidative damage via the determination of malondialdehyde (MDA) concentration. All samples were loaded in triplicate to account for technical errors. A spectrophotometer microplate reader was used to determine the absorbance readings for all the oxidative stress assays indicated.

### 2.7 Western blotting

Sample homogenates designated for Western blotting were prepared in a ratio of 1:9 with radioimmunoprecipitation assay lysis buffer. Samples were pooled according to experimental grouping (Martin et al., 2008). Specific details on blocking and antibody conditions, as well as product numbers are listed in Table 2. Appropriate protein concentrations were prepared with 2× Laemmli buffer solution and electrophoresed in various SDS‐PAGE gels with total protein used as a loading control. Gels were activated under UV light using the Chemidoc TM MP Gel Imaging system (Bio‐Rad Laboratories Inc., Hercules, CA, USA), transferred onto low fluorescence PVDF membranes (7 min at 2.5 A, 25 V using the Trans‐Blot® Turbo™ semi‐dry transfer system; Bio‐Rad Laboratories Inc.), and transfer efficiency was assessed before blocking. Membranes were blocked at room temperature then incubated with primary antibodies (Table 2)—overnight at 4°C. Membranes were further incubated for 1 h at room temperature with HRP‐conjugated goat anti‐rabbit (G21234; Thermo Fisher Scientific, Waltham, MA, USA) secondary antibody at a ratio of 1:10,000. Membranes were captured (ChemiDoc TM MP Imaging System, Bio‐Rad Laboratories Inc.) and bands quantified using ImageLab 6 analysis software (Image Lab Software, Bio‐Rad Laboratories Inc.). Data were normalized against the total lane protein of each sample in conjunction with a reference standard containing protein from each sample. Total lane protein images were acquired using the stain‐free blot imaging tool in the ImageLab 6 analysis software. Data were analyzed in such a manner that all group protein relative expression values were converted into the relative expression to the control group average.

**TABLE 2:** Primary antibody details and blocking conditions for all proteins of interest.

Protein	% gel	Blocking buffer	Blocking time	1°Ab	[1°Ab]
SOD1	15%	5% Fat‐free milk	1 h	ab16831, Abcam	1:1000
SOD2	15%	5% Fat‐free milk	1 h	ab68155, Abcam	1:1000

### 2.8 Statistical analyses

All statistical analyses were conducted using GraphPad Prism 7.04 (GraphPad Software Inc., La Jolla, CA, USA). Data were subject to normality testing (Shapiro–Wilks) as well as outlier detection (Grubbs test, α = 0.05). Significance between groups was determined through various tests depending on the distribution of the data as well as variance. An unpaired Student's *t*‐test or a Mann–Whitney test was applied to parametric data and nonparametric data respectively. Bonferroni–Dunn post hoc tests confirmed significance and accounted for multiple comparisons. *p*‐value less than or equal to 0.05 was considered significant. Data are presented as mean ± standard deviation (SD).

## 3 RESULTS

### 3.1 UCMS model

**FIGURE 1:**
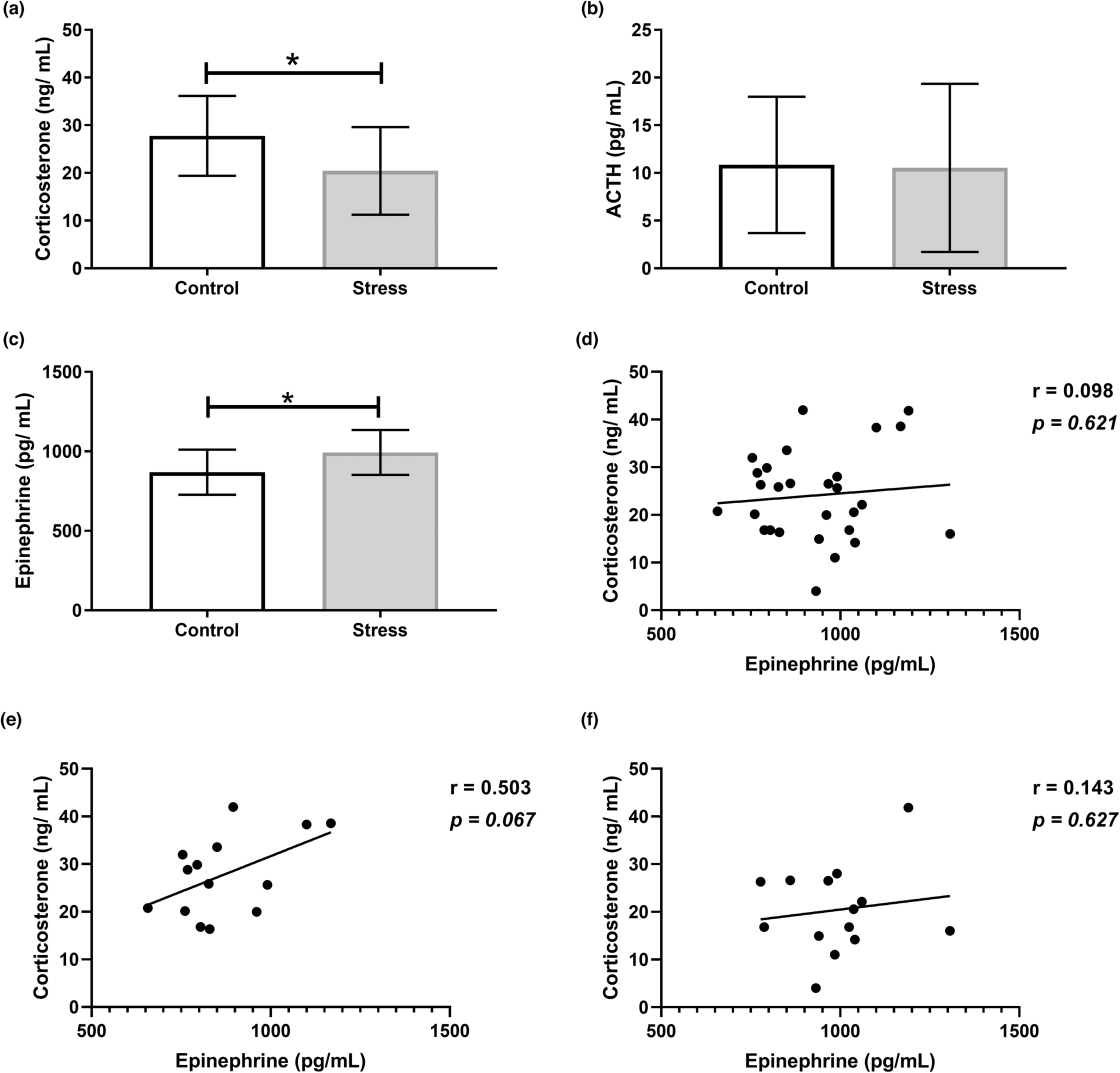
UCMS altered indicators of both the HPA axis and SAM pathway. (a) Plasma corticosterone; (b) plasma ACTH; (c) plasma epinephrine; (d) correlation between corticosterone and epinephrine (control and stress groups); (e) corticosterone vs epinephrine in control animals only; (f) corticosterone vs epinephrine in stress animals only. Data expressed as mean ± standard deviation. *n* = 14 per group. *p < 0.05.

Our data revealed increased epinephrine (*p* < 0.05; Figure 1a) and decreased corticosterone plasma levels (*p* < 0.05; Figure 1b) in the Stress cohort versus Controls. However, plasma ACTH levels did not differ significantly between groups (Figure 1c).

### 3.2 Organ‐specific redox status

**FIGURE 2:**
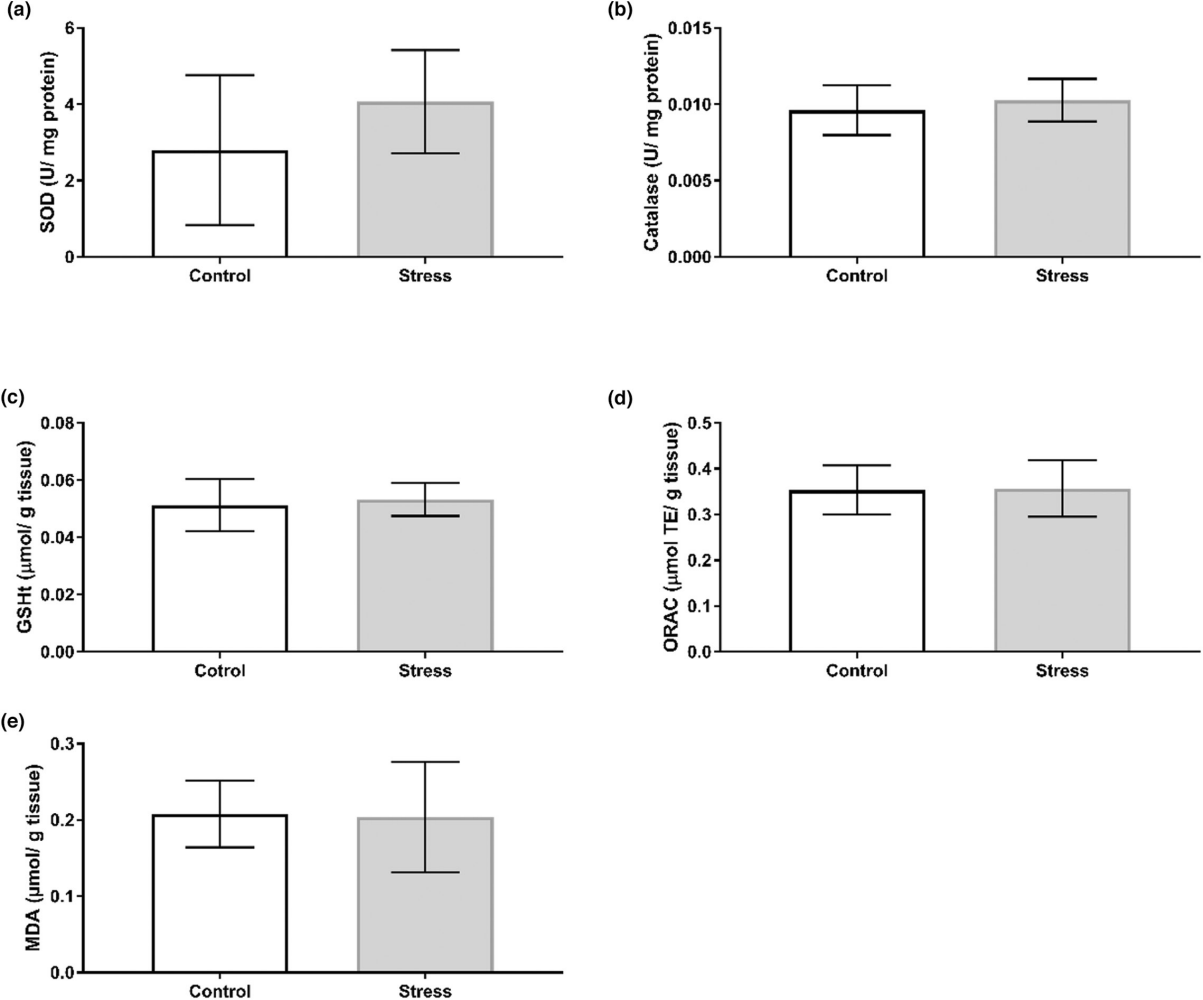
Assessment of hippocampal redox status in response to chronic stress. (a) Superoxide dismutase activity (Control; *n* = 10; Stress *n* = 11); (b) catalase activity (Control: *n* = 11; Stress: *n* = 12); (c) total GSH (Control: *n* = 11; Stress: *n* = 12); (d) ORAC (Control: *n* = 11; Stress: *n* = 12); (e) MDA formation (Control: *n* = 11; Stress: *n* = 12). Data presented as mean ± standard deviation.

**FIGURE 3:**
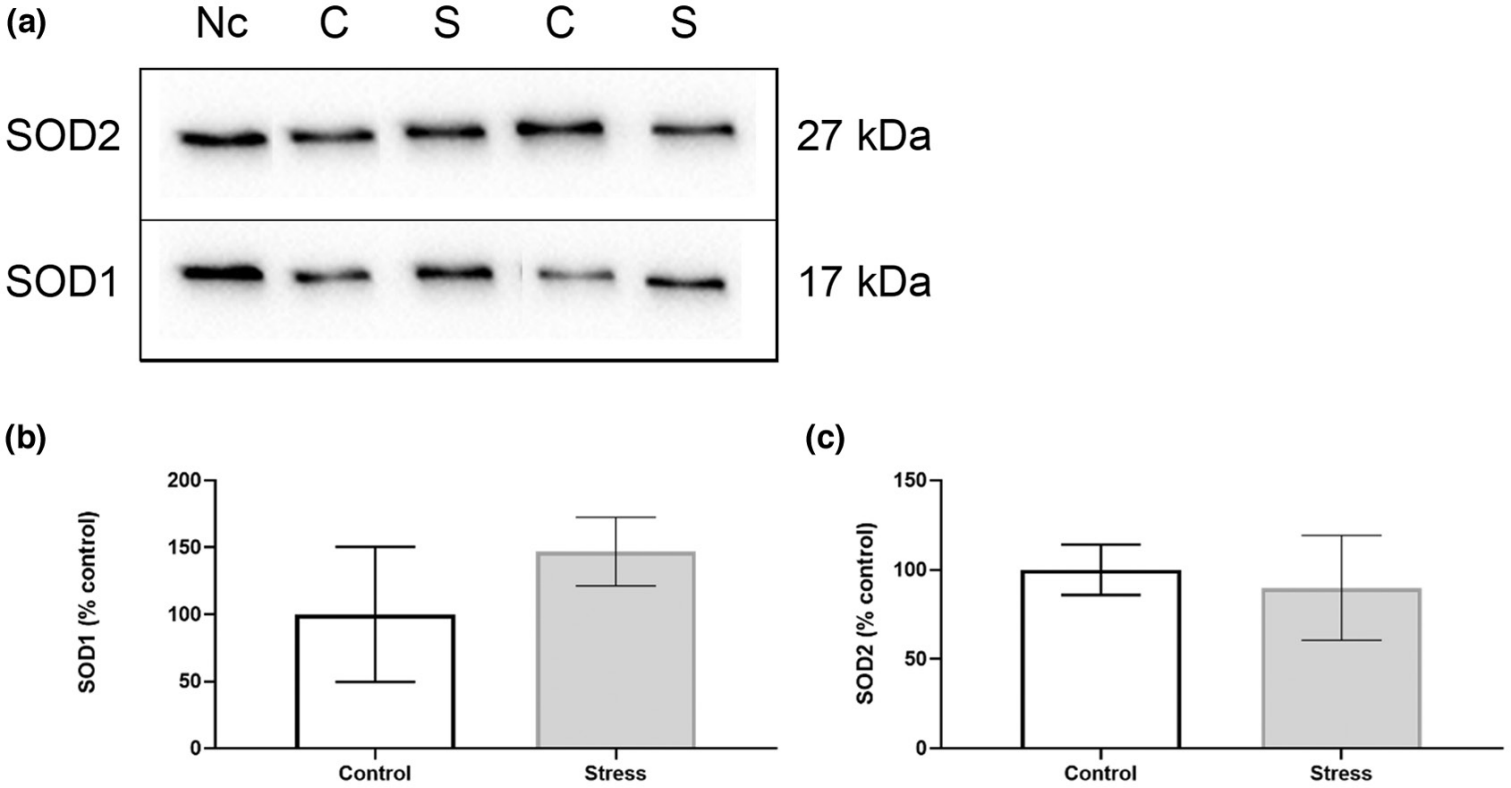
Western blotting analyses of hippocampal SOD1/2 expression. (a) Representative chemiluminescent image of SOD1 and SOD2 within hippocampal samples. (b) Quantification of protein bands for SOD1 and (c) SOD2. Data presented as a percentage of control mean ± standard deviation (*n* = 12 per group). Nc, normalization control; C, control group; S, stress group.

**FIGURE 4:**
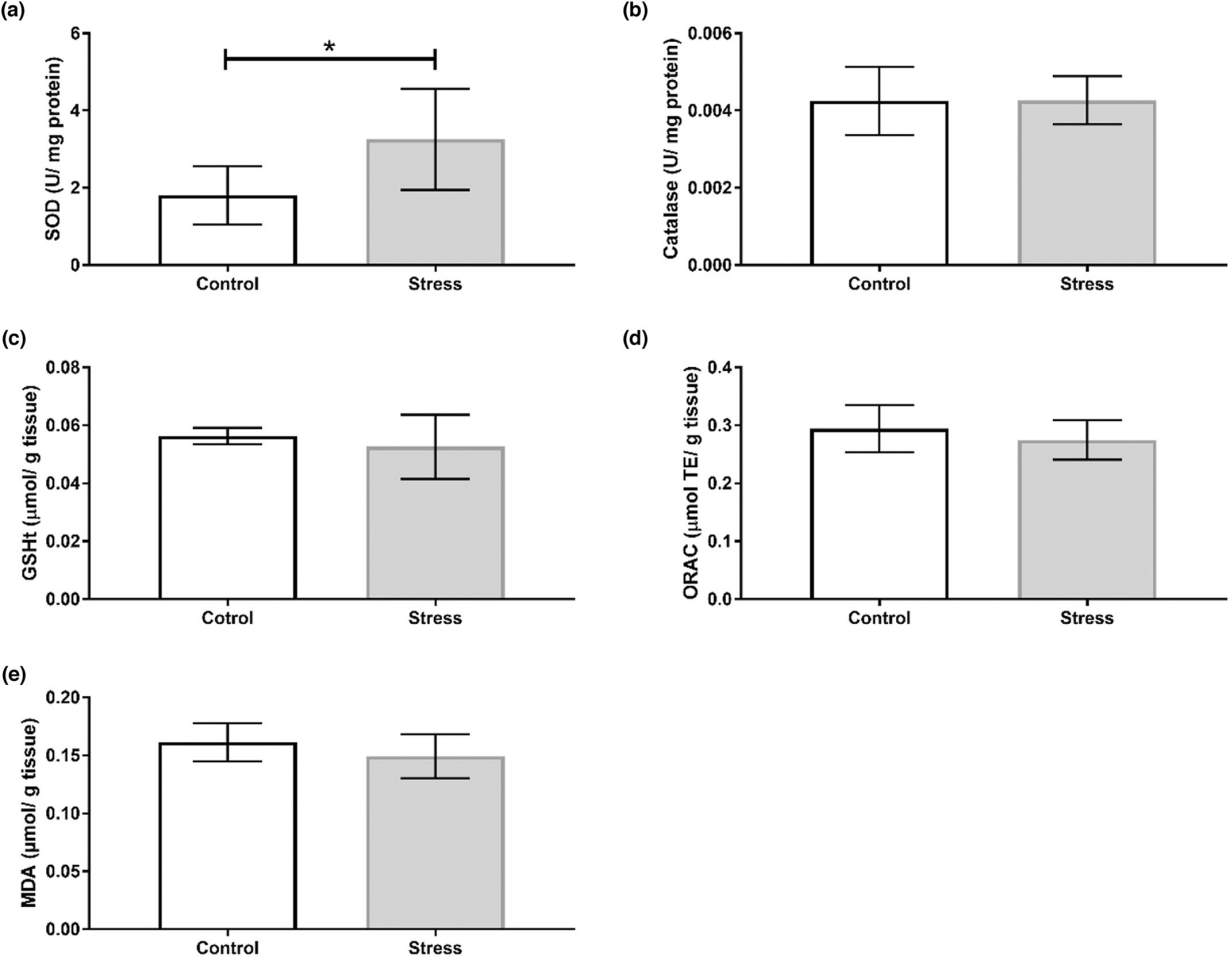
Assessment of prefrontal cortex redox status in response to chronic stress. (a) Superoxide dismutase activity (Control: *n* = 8; Stress: *n* = 9); (b) catalase activity (Control: *n* = 10; Stress: *n* = 11); (c) total GSH (Control: *n* = 10; Stress: *n* = 11); (d) ORAC (Control: *n* = 10; Stress: *n* = 11); (e) MDA formation (Control: *n* = 10; Stress: *n* = 11). Data presented as mean ± standard deviation. *p < 0.05.

**FIGURE 5:**
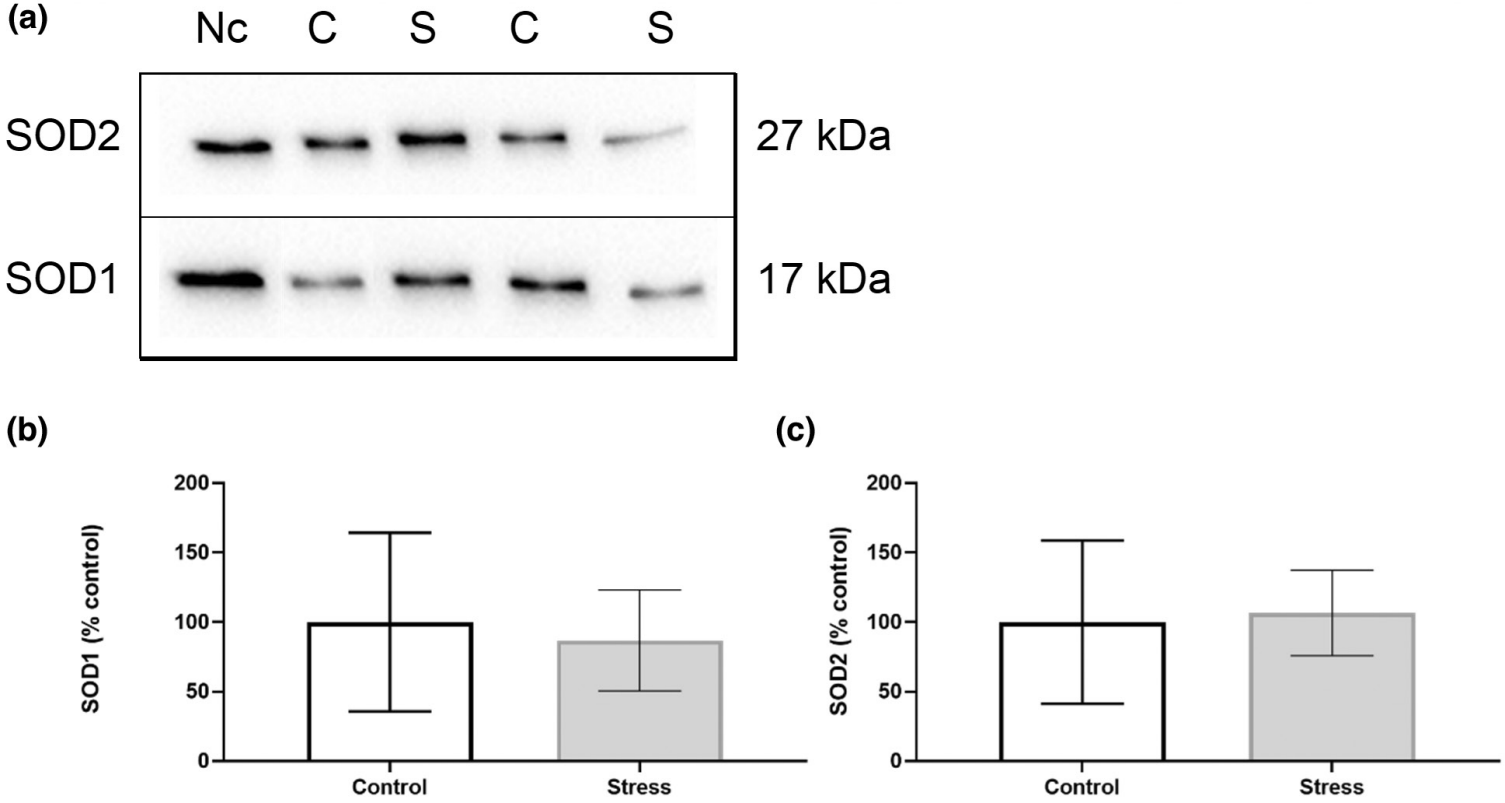
Western blotting analyses of prefrontal cortex SOD1/2 expression. (a) Representative chemiluminescent image of SOD1 and SOD2 within prefrontal cortex samples. (b) Quantification of protein bands for SOD1 and (c) SOD2. Data presented as a percentage of control mean ± standard deviation (*n* = 12 per group). Nc, normalization control; C, control group; S, stress group.

**FIGURE 6:**
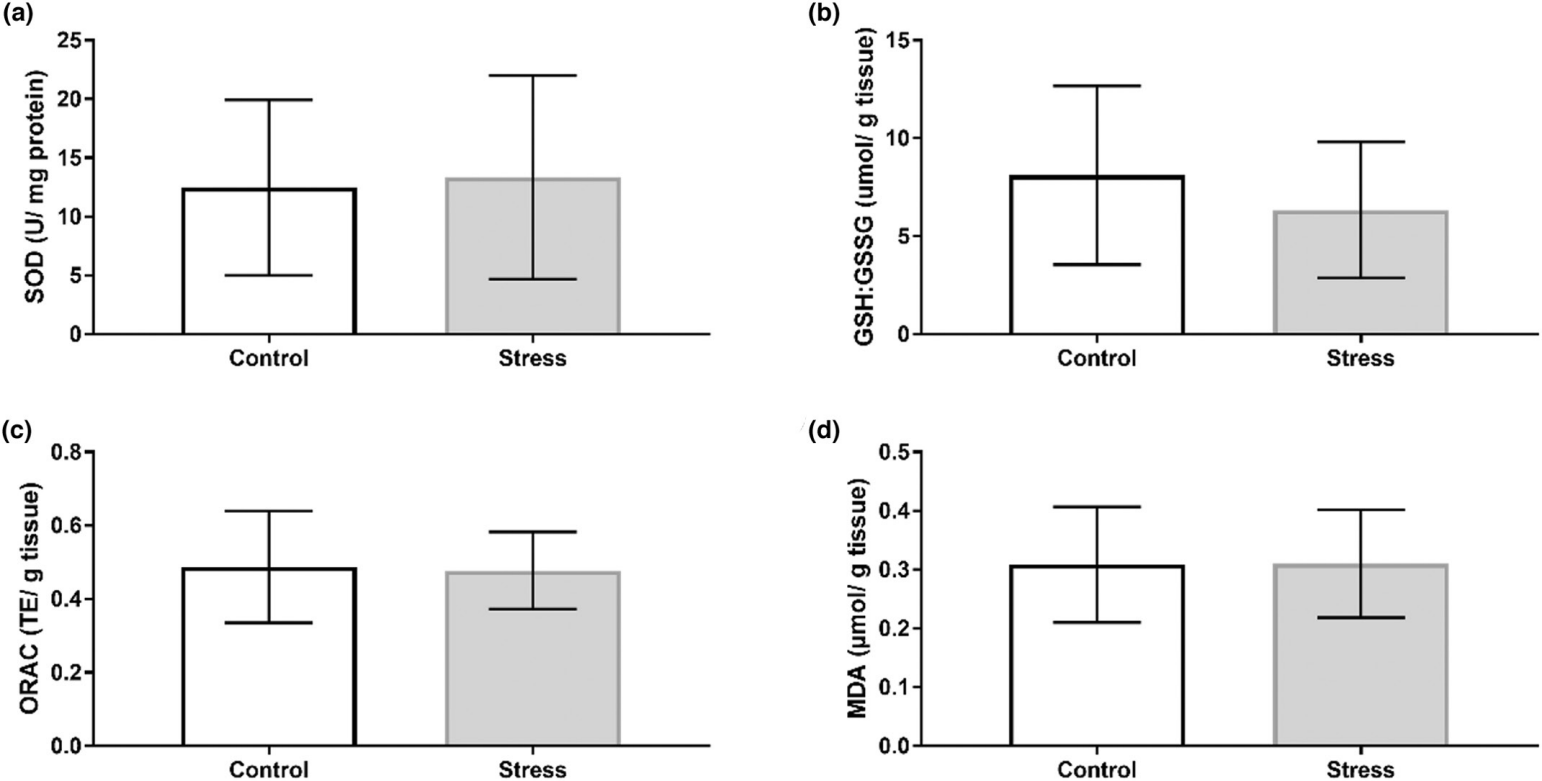
Assessment of cerebellar redox status in response to chronic stress. (a) Superoxide dismutase activity; (b) GSH:GSSG ratio; (c) ORAC; (d) MDA formation. Data presented as mean ± standard deviation. *n* = 12 per group.

**FIGURE 7:**
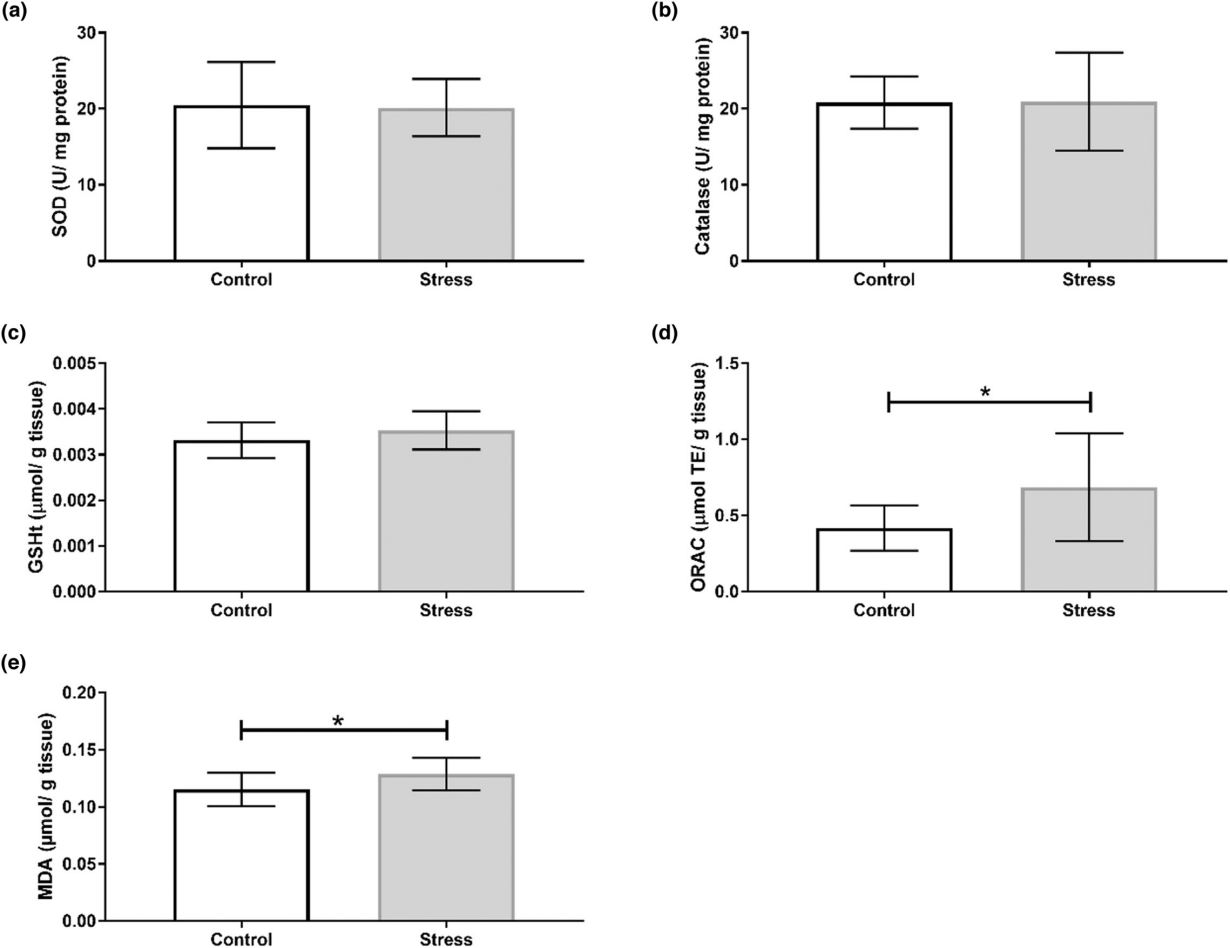
Assessment of cardiac redox status in response to chronic stress. (a) Superoxide dismutase activity (*n* = 12/ group); (b) catalase activity (*n* = 12/group); (c) total GSH (*n* = 12/group); (d) ORAC (*n* = 12/group); (e) MDA formation (*n* = 11/group). Data presented as mean ± standard deviation. *p < 0.05.

**FIGURE 8:**
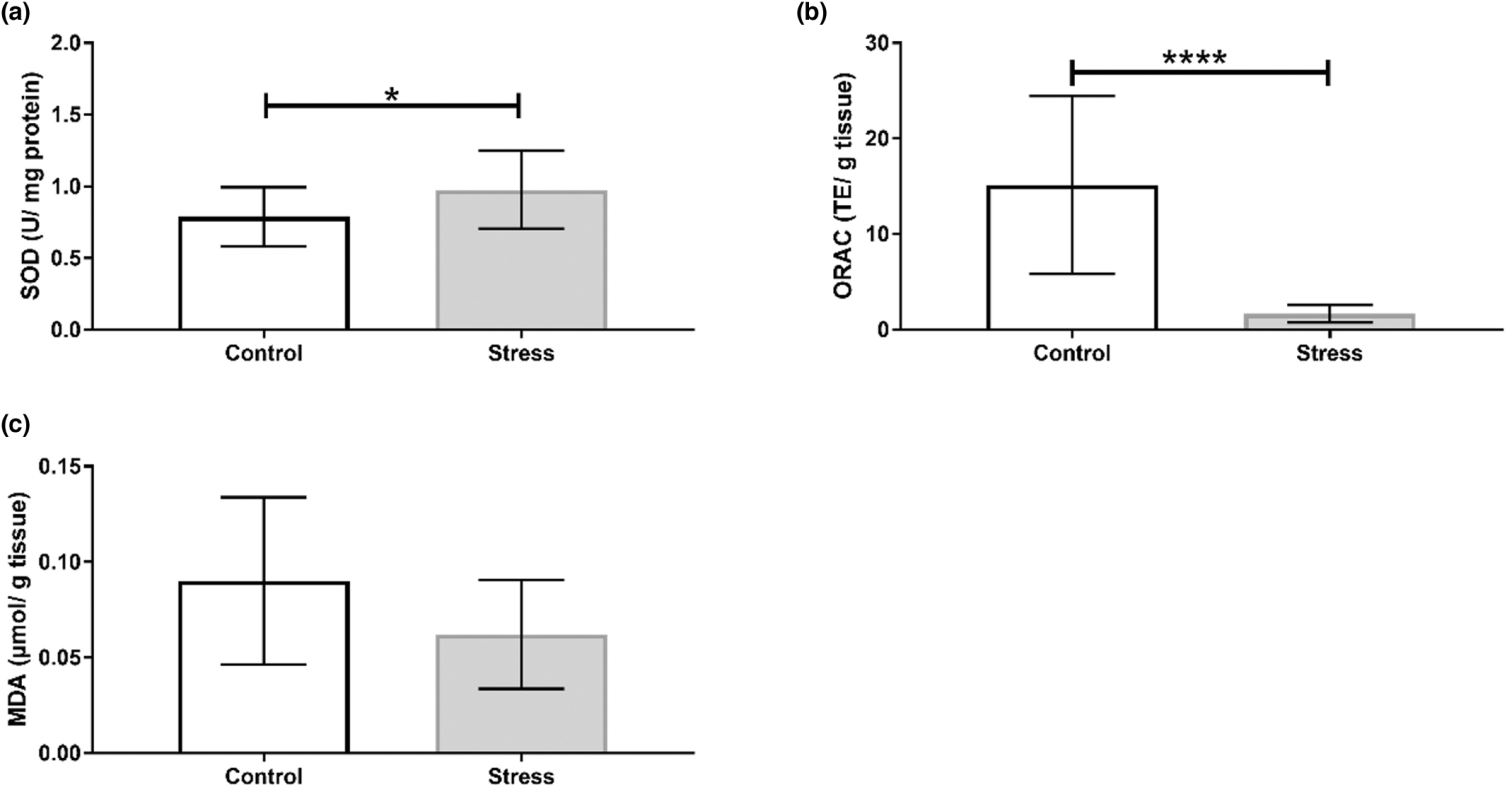
Assessment of hepatic redox status in response to chronic stress. (a) Superoxide dismutase activity; (b) ORAC; (c) MDA formation. *n* = 14/ group. Data presented as mean ± standard deviation. *p < 0.05; ****p < 0.001.

Our findings show that the UCMS protocol induced organ‐specific oxidative perturbations (Table 3). No alterations to antioxidant activity nor capacity were observed in the hippocampus (Figure 2), with no changes in SOD isoform expression (Figure 3). Oxidative stress assays show that SOD activity increased in the prefrontal cortex of the Stress cohort (*p* < 0.05; Figure 4a). However, Western blotting analyses revealed no significant changes in SOD1 or SOD2 expression between Control and experimental groups in the prefrontal cortex (Figure 5). The cerebellum displayed a similar redox profile to that of the hippocampus with no significant alterations to antioxidant capacity nor activity (Figure 6). Aside from enzymatic antioxidant activity, our data indicate the UCMS model increased cardiac antioxidant capacity (*p* < 0.05; Figure 7D). This was accompanied by a small increase in SOD activity (*p* < 0.05; Figure 8a), while simultaneously lowering nonenzymatic antioxidant capacity (*p* < 0.001; Figure 8b) within the liver of the Stress cohort. The presence of oxidative damage was assessed through MDA formation. Only the cardiac tissue of the Stress cohort indicated mild oxidative damage through elevated MDA levels (*p* < 0.05; Figure 7e).

**TABLE 3:** Comparison of endogenous tissue antioxidant defense system activity and oxidative damage markers in control and stress samples.

SOD activity (U/mg protein)	Control	Stress
HC	2.801 ± 1.966****	4.072 ± 1.349****
PFC	**1.801 ± 0.752** ****	**3.258 ± 1.310** ***
CER	12.480 ± 7.450****	14.730 ± 7.520**
Heart	20.480 ± 5.657	20.150 ± 3.755
Liver	**0.788 ± 0.207** ****	**0.977 ± 0.270** ****
CAT activity (U/mg protein)		
HC	0.001 ± 0.002****	0.010 ± 0.001****
PFC	0.004 ± 0.001****	0.004 ± 0.001****
Heart	20.820 ± 3.435	20.950 ± 6.430
ORAC (TE/g tissue)		
HC	0.354 ± 0.054	0.357 ± 0.061
PFC	0.294 ± 0.041	0.275 ± 0.034
CER	0.487 ± 0.152	0.478 ± 0.105
Heart	**0.418 ± 0.149**	**0.686 ± 0.354**
Liver	**15.140 ± 9.300** ****	**1.666 ± 0.915**
MDA (μmol/g tissue)		
HC	0.208 ± 0.044****	0.204 ± 0.072**
PFC	0.161 ± 0.016	0.149 ± 0.019
CER	0.309 ± 0.099****	0.310 ± 0.092****
Heart	**0.115 ± 0.015**	**0.129 ± 0.014**
Liver	0.090 ± 0.044	0.062 ± 0.029**

*Note*: Compared with reference tissue (heart) group. Bold values indicate a significant difference between control and stress groups within each tissue type.

** *p* < 0.01

*** *p* < 0.001

**** *p* < 0.0001.

## 4 DISCUSSION

The UCMS protocol elicited a mildly anxious phenotype versus Control rats. Our data revealed distinct organ‐specific vulnerability to redox perturbations following the stress protocol and may provide unique insights into future therapeutic targets.

### 4.1 UCMS and the stress response

The activation of the stress response increases the release of HPA axis and SAM pathway mediators and hence a corresponding rise in their circulating concentrations would be expected following chronic stress (Kay et al., 2020). Although the SAM pathway and HPA axis work independently of one another, positive crosstalk does exist and therefore corticosterone and epinephrine are positively correlated in a well‐functioning physiological system (Dickerson & Eichenbaum, 2010; Geng et al., 2016; Jeong et al., 2015; Schneiderman et al., 2005; Seeley, 2019). This relationship led to the widespread adoption of elevations in corticosterone and catecholamines as the primary biological determinant of whether a model successfully induced stress (Noushad et al., 2021). However, disruptions in such crosstalk can occur in response to chronic stress, indicating dysfunction (Kokkinopoulou & Moutsatsou, 2021; Langlais et al., 2012). Dividing the correlation analyses between corticosterone and epinephrine into Control and Stress groups yielded an interesting finding. Here, the Control group displayed a strong positive correlation between corticosterone and epinephrine (Figure 1e) which was abolished in the Stress cohort (Figure 1f). This further highlights the disruption in crosstalk between the HPA axis and SAM pathway in our Stress cohort. The significant increase in plasma epinephrine together with decreased corticosterone levels therefore suggests that our UCMS protocol was sufficient to induce a phenotype of chronic stress.

The UCMS is widely reported as a model of major depression and as such, behavioral changes constitute an important marker of model efficacy (Evanson et al., 2010; Gao et al., 2020). Our study therefore employed the elevated plus maze as a means of assessing anxious behavior within the rats (Brose, 2021). Data showed increased stretch‐attend posture in the stress group—a risk assessment behavior associated with mild anxiety (Kinlein et al., 2015). Here, a lowered back, elongated body, and cautious forward movement indicate fearful risk assessment and hesitancy to explore (Holly et al., 2016). This behavior is in direct opposition to an unstressed rodent's tendency to explore novel environments (Walf & Frye, 2007). Taken together with the CORT and EPI results, this indicates that our protocol induced a mild anxiety‐like phenotype rather than the typical depressive state associated with this model (Evanson et al., 2010). This illustrates the heterogeneity of stress‐related disorders and the importance of including various stress markers regardless of the expected clinical endpoint. Hence, failure to include a broad range of markers and the sole reliance on expected changes in CORT and EPI levels can result in false negatives and a lack of insight regarding the various stages and presentations of chronic stress.

### 4.2 Organ‐specific redox perturbations

Research suggests that oxidative stress is a significant driver of stress‐related pathology in multiple organs and can impact intracellular bioenergetics, structural integrity, and ultimately survival of the organism (Harris, 2015; McEwen, 2009). Although substantial evidence supports the influence of chronic stress on organ redox homeostasis, limited studies investigate more than one organ simultaneously. Moreover, the degree by which chronic stress alters organ‐specific redox balance depends on the intensity, duration, and type of stressor utilized in the model (Hussien et al., 2019a; López‐López et al., 2016; Sahin et al., 2006; Sood et al., 2006). Of note, an increase in enzyme activity or expression generally indicates an adaptive response to combat excess free radicals linked to the early stages of stress (Alkadhi, 2013). Our analyses yielded some interesting findings that further highlight the differential organ susceptibility to the effects of chronic stress (Table 3).

### 4.3 Brain region redox perturbations

The brain is a lipid‐rich and highly oxidative organ and is thus noticeably susceptible to oxidative damage (Shichiri, 2014). An imbalance between pro‐ and antioxidants is therefore an attractive theory of neurological dysfunction that is supported by strong evidence (Liu et al., 2015; Miller & Sadeh, 2014; Patel, 2016; Smith et al., 2000). However, it is important to note that the different neural regions exhibit varying antioxidant capacities in relation to one another (Kamsler & Segal, 2003; Thiels & Klann, 2002; Wang et al., 2005). Our Control animal results illustrate this with an equivalent nonenzymatic antioxidant capacity across all brain regions and the cerebellum containing the most SOD activity. The cerebellum displayed significantly elevated MDA, showing the greatest degree of lipid peroxidation of all tissues analyzed in the absence of stress.

When comparing these parameters in the stress cohort, a similar pattern is observed although the degree of significance is altered. However, only the prefrontal cortex of Stressed rats revealed a significant increase in SOD activity compared with Controls, despite a lack of changes in overall antioxidant capacity nor any indication of increased oxidative‐induced lipid damage. Moreover, the redox changes observed in the prefrontal cortex region were not reflected in the hippocampus or the cerebellum. The Western blotting data suggest that such an increase in SOD activity is likely due to post‐translational modifications and not due to increased expression levels (Bouayed & Soulimani, 2019; Díaz‐Cruz et al., 2007, 2011; Fisher, 2009; Maher & Schubert, 2000; Vilchis‐Landeros et al., 2020; Wang et al., 2018). This could suggest an adaptive mechanism at play in the prefrontal cortex in response to increased signaling demands. Despite their notoriety as destructive molecules, O_2_^•‐^ and H_2_O_2_ also serve as important signaling molecules within the brain through the oxidation of redox‐sensitive cysteine residues on signaling proteins (Barford, 2004; van der Reest et al., 2018). Known signaling targets of H_2_O_2_ include protein phosphatases, nonreceptor protein tyrosine kinases, protein kinase C, mitogen‐activated protein kinases, and transcriptional factors such as Nrf and Nuclear factor kappa‐light‐chain‐enhancer of activated B cells (Antunes & Brito, 2017; di Marzo et al., 2018) SODs therefore play a dual role as ROS signalers and scavengers (Wang et al., 2018).

Together, the UCMS protocol induced mild changes in the redox status of only the prefrontal cortex, indicating differential susceptibility to oxidative stress between the regions under investigation under stress conditions (Bremner et al., 2000; Rosso et al., 2005; Vakili et al., 2000).

#### 4.3.1 Mild cardiac oxidative damage

The heart is a highly metabolic organ with extensive energy demands. Oxidation of a number of fuel substrates ensures a constant supply of ATP for cardiac contractility and ion gradient regulation (Lopaschuk & Ussher, 2016). Increased heart rate, ejection fraction, and blood pressure are all hallmarks of a stress response and stem from increased cardiac work (Chu et al., 2022). Increased work requires heightened energy production, which coincides with elevated ROS. Due to its limited regenerative capabilities, the heart relies heavily on endogenous antioxidant defenses for protection against oxidative stress, a key component of cardiovascular disease initiation and progression (Lopaschuk & Ussher, 2016; Xu et al., 2019b). In support, the cardiac tissue of both Control and Stress groups displayed the greatest activity of enzymatic antioxidants (i.e., SOD and CAT) compared with the other organs. The heart is thus primed to adapt to stress stimuli and counteract any overproduction of ROS as a byproduct of unregulated metabolism. However, prolonged exposure to stress hormones potentiates lipid peroxidation, which generally occurs due to an overabundance of reactive oxygen and nitrogen species and/or diminished antioxidant defense systems (Hussien et al., 2019b). As such, the presence of elevated MDA levels *together* with upregulated ORAC in the heart tissue of the Stress cohort, was an unexpected result. This combination of results has not been observed in preclinical chronic stress research in the heart.

Total nonenzymatic antioxidant capacity is rarely investigated in the heart, particularly in the context of chronic stress. Our results suggest that cardiac non‐enzymatic antioxidant capacity was upregulated in the heart to counteract rising oxidative stress, and to thereby attenuate MDA formation. Vitamin E is abundant in the cytosol and mitochondrial membranes where it scavenges free radicals and mitigates lipid peroxidation initiation (Ferrari et al., 1991). However, we postulate that this adaptive mechanism was insufficient to completely prevent lipid peroxidation, and thus, we were able to detect an increase in response to chronic stress.

The associations between MDA and pathology are numerous with increased concentrations observed in neurological, cardiometabolic diseases, and cancers. (Ayala et al., 2014; Cristalli et al., 2012; Niedernhofer et al., 2003; Wang et al., 1996). Increased cardiac MDA levels have been found in response to norepinephrine administration and exposure to certain rodent chronic stress models (Hu et al., 2020; Neri et al., 2007). While chronic overexposure to catecholamines is a likely source of ROS and the primary contributor to lipid peroxidation in our UCMS model, corticosterone can also contribute to oxidative stress via inflammatory signaling (Adameova et al., 2009; Costa et al., 2012; Kunz‐Ebrecht et al., 2003; Rutledge et al., 2013; Tappia et al., 2001). Although corticosterone normally exerts trans‐repressive effects on proinflammatory cytokine production during a normal stress response (Petta et al., 2016), the Stress cohort displayed decreased plasma levels. This suggests that the anti‐inflammatory effects of corticosterone may have been attenuated in the Stress cohort, which could possibly result in an inflammatory response that induces oxidative damage.

Taking all the above into account, our findings suggest that even though the Stress group hearts antioxidant activities were equal to (i.e., SOD and CAT), and greater (i.e., ORAC) to that of the control group, mild oxidative damage still occurred. Due to the transient, dynamic nature of redox homeostasis, the Stress cohort may still be within the adaptive phase of chronic stress.

#### 4.3.2 Hepatic antioxidant capacity and activity

Although there is a large body of evidence supporting the role of chronic stress in the development of various pathologies (Cohen et al., 2012; Sher et al., 2020), relatively little is known in terms of the effects of chronic stress on the liver. Our findings from our Control animals highlight the hepatic tissue's inherent abundance of nonenzymatic antioxidants and relatively low levels of oxidative damage. However, the UCMS protocol severely decreased overall nonenzymatic antioxidant capacity in livers isolated from the stressed rats, yet no alterations to lipid peroxidation were observed. The marked increase in SOD activity in the Stress rats is consistent with its role as the first line of defense against free radicals and helps to explain the lack of changes observed in terms of lipid peroxidation (Ighodaro & Akinloye, 2018). These data show that the liver is a relatively resilient organ within the context of UCMS‐induced oxidative stress.

### 4.4 Conclusion

The UCMS protocol used in this study induced a mildly anxious phenotype in the Stress/ Stressed group. This caused consequent downstream perturbations to the SOD activity of the prefrontal cortex and liver, as well as changes to cardiac antioxidant capacity and prooxidant load. The lack of damage markers in the prefrontal cortex and liver, in combination with the observed increased SOD activity, suggests that these two tissues have a higher resilience to redox stress than the heart, when viewed in the context of a stress‐induced anxiety phenotype. This indicates that the heart is more susceptible to oxidative stress under stressed conditions, and that further research is warranted in order to limit the onset of stress‐induced oxidative stress.

## AUTHOR CONTRIBUTIONS

Hannah Geddie, Megan Cairns, Leandrie Beselaar, Nina Truter, and M. Faadiel Essop designed the study. Hannah Geddie, Megan Cairns, Logan Smith, Minette van Wyk, Leandrie Beselaar, Nina Truter, and Fanie Rautenbach performed the experiments. Hannah Geddie, Megan Cairns, Logan Smith, Minette van Wyk, Leandrie Beselaar, Nina Truter, Fanie Rautenbach, Jeanine L Marnewick, Danzil E. Joseph, and M. Faadiel Essop analyzed the data and prepared the figures. Hannah Geddie, Megan Cairns, Leandrie Beselaar, Nina Truter, and M. Faadiel Essop drafted the manuscript. All authors edited and revised the manuscript. All authors have read and approved the final version of the manuscript.

## CONFLICT OF INTEREST STATEMENT

The authors confirm that there is no conflict of interest.
